# Pharmacokinetic Profiling of Ginsenosides, Rb1, Rd, and Rg3, in Mice with Antibiotic-Induced Gut Microbiota Alterations: Implications for Variability in the Therapeutic Efficacy of Red Ginseng Extracts

**DOI:** 10.3390/foods12234342

**Published:** 2023-12-01

**Authors:** Jeon-Kyung Kim, Min Sun Choi, Hee-Seo Park, Kyung Hwa Kee, Dong-Hyun Kim, Hye Hyun Yoo

**Affiliations:** 1 Neurobiota Research Center, College of Pharmacy, Kyung Hee University, Dongdaemun-gu, Seoul 02447, Republic of Korea; jkkim@jbnu.ac.kr (J.-K.K.); xlvksl1997@khu.ac.kr (H.-S.P.); 2 School of Pharmacy, Institute of New Drug Development, Jeonbuk National University, Jeonju 54896, Republic of Korea; 3 Pharmacomicrobiomics Research Center, College of Pharmacy, Hanyang University, Ansan 15588, Republic of Korea; mschoi@kirams.re.kr (M.S.C.); khkee9202@gmail.com (K.H.K.)

**Keywords:** red ginseng, ginsenoside, metabolism, gut microbiota

## Abstract

Ginsenoside Rg3 is reported to contribute to the traditionally known diverse effects of red ginseng extracts. Significant individual variations in the therapeutic efficacy of red ginseng extracts have been reported. This study aimed to investigate the effect of amoxicillin on the pharmacokinetics of ginsenosides Rb1, Rd, and Rg3 in mice following the oral administration of red ginseng extracts. We examined the α-diversity and β-diversity of gut microbiota and conducted pharmacokinetic studies to measure systemic exposure to ginsenoside Rg3. We also analyzed the microbiome abundance and microbial metabolic activity involved in the biotransformation of ginsenoside Rb1. Amoxicillin treatment reduced both the α-diversity and β-diversity of the gut microbiota and decreased systemic exposure to ginsenoside Rg3 in mice. The area under the curve (AUC) values for Rg3 in control and amoxicillin-treated groups were 247.7 ± 96.6 ng·h/mL and 139.2 ± 32.9 ng·h/mL, respectively. The microbiome abundance and microbial metabolic activity involved in the biotransformation of ginsenoside Rb1 were also altered by amoxicillin treatment. The metabolizing activity was reduced from 0.13 to 0.05 pmol/min/mg on average. Our findings indicate that amoxicillin treatment potentially reduces the gut-microbiota-mediated metabolism of ginsenoside Rg3 in mice given red ginseng extracts, altering its pharmacokinetics. Gut microbiome variations may thus influence individual ginsenoside pharmacokinetics, impacting red ginseng extract’s efficacy. Our results suggest that modulating the microbiome could enhance the efficacy of red ginseng.

## 1. Introduction

Red ginseng, obtained from the root of Panax ginseng Meyer through the process of steaming and drying, is widely available as a functional food in Asian countries [1,2,3]. Ginseng has been popularly used to promote longevity and health for over 2000 years in Asian cultures [4]. In addition to ancient texts suggesting that the long-term consumption of ginseng may help extend life, a recent study analyzed the correlation between ginseng consumption and cause-specific mortality, identifying a significant association with reduced all-cause mortality [5,6]. Furthermore, experimental studies have provided evidence of the potential therapeutic effects of Panax ginseng, including its anticancer, cardioprotective, and neuroprotective properties, and over the past 20 years in developed countries, clinical studies have shown benefits for diabetes, cardiovascular disorders, cognition, recall, mood, and cold and flu. Additionally, positive effects have been observed in cancer-related fatigue, overall well-being, life satisfaction, and social functioning, among other aspects [7,8,9,10].

Ginsenoside, a triterpene saponin, is a distinctive component of ginseng with multiple pharmacological activities such as anti-inflammatory and anti-cancer effects, the inhibition of platelet aggregation, anti-aging properties, and anti-diabetic activity [11,12,13,14]. Among the main components of red ginseng, the ginsenosides Rb1, Rb2, Rc, Rd, and Rg3 stand out, with Rb1 being the most abundant [15]. The gut microbiota is pivotal in the metabolism of polar ginsenosides such as Rb1, Rb2, and Rc, converting them into non-polar ginsenosides such as Rd and Rg3, which are then absorbed into the bloodstream. For instance, ginsenoside Rb1 undergoes sequential cleavage of glycosidic bonds by the gut microbiota, leading to the production of ginsenosides Rd, F2, Rg3, compound K, and protopanaxadiol [16]. Therefore, the metabolism and biotransformation of ginsenosides within the gastrointestinal tract are influenced through the actions of the gut microbiota [17,18]. Consequently, alterations in the gut microbiota can modulate the metabolism and absorption of ginsenosides, ultimately impacting their bioavailability and biological activities.

The gut microbiota consists of numerous microorganisms that inhabit the digestive tract [19]. These microorganisms play a crucial role in metabolizing xenobiotics, including drugs, food, and natural products, prior to their absorption into the bloodstream [20]. When antibiotics are administered orally, they can decrease the diversity of microbiota species and disturb the makeup of the gut microbiota, subsequently influencing the functions of gut microbial enzymes. Consequently, oral antibiotic treatment can potentially induce an altered microbial metabolism of xenobiotics, including drugs and phytochemicals. This suggests that oral antibiotics may also impact the microbial metabolism of ginsenosides in the gut, leading to potential pharmacokinetic interactions between the two. However, there is still limited research on the interactions between antibiotics and ginsenosides.

This study investigated the effects of the antibiotic amoxicillin on ginsenoside gut-microbiota-mediated pharmacokinetics in mice. Ginsenoside Rg3 is recognized as a key component in red ginseng, renowned for its pharmacological efficacy and diverse physiological activities, including immune modulation, neuroprotective effects, and anti-cancer properties across various diseases and cancer types [21,22]. Ginsenoside Rg3 originates from the gut-microbiota-mediated biotransformation of polar ginsenosides including Rb1, Rb2, and Rc [23,24]. Accordingly, changes in the biotransformation of these polar ginsenosides by the microbiome are anticipated to influence the levels of ginsenoside Rg3 absorbed into the body, potentially impacting the efficacy of red ginseng. Our study particularly focused on exploring the alterations in the pharmacokinetics of ginsenoside Rg3 following alterations in the gut microbiota due to antibiotic administration.

## 2. Materials and Methods

### 2.1. Materials 

The red ginseng extract was supplied by the Korea Ginseng Institute (Daejeon, Republic of Korea). The contents of ginsenosides Rb1, Rd, and Rg3 in the red ginseng extract were 16.1 mg/g, 3.2 mg/g, and 0.7 mg/g, respectively. The HPLC chromatogram for the chemical fingerprint is provided as a Supplementary File (Figure S1). Ginsenosides Rb1, Rd, and Rg3 were purchased from ChemFaces (Wuhan, China). Digitoxin (internal standard) and amoxicillin were purchased from Sigma-Aldrich (St. Louis, MO, USA). Acetonitrile, methanol, water, and formic acid were purchased from J.T. Baker (Phillipsburg, NJ, USA). All other chemicals utilized were in accordance with analytical quality.

### 2.2. Animals

Six-week-old male C57BL/6 mice (18–23 g) were obtained from the Orient Experimental Animal Breeding Center (Gyeonggi-do, Republic of Korea). The mice were kept in a facility under controlled environmental conditions, with a fixed temperature (25 ± 2 °C) and humidity (55 ± 10 °C) and a 12 h light/dark cycle. The execution of all animal procedures complied with the Guide for the Care and Use of Laboratory Animals (National Institutes of Health) and the Hanyang University Guides for Laboratory Animal Care and Use and were approved by the Committee for the Care and Use of Laboratory Animals in the College of Pharmacy, Hanyang University (2016-0151; 15 July 2016). 

### 2.3. Pharmacokinetic Experiments

The mice were randomized into two groups: control and amoxicillin-treated mice. The mice were orally administered either a PBS buffer (pH 7.4) or amoxicillin (250 mg/kg) for five consecutive days. Pharmacokinetic experiments were conducted on the second day following the last antibiotic administration. Red ginseng was administered orally (10 g/kg), and blood samples were taken from the orbital vein into heparinized tubes at 30 min, followed by at 1, 2, 4, 6, 9, 12, and 24 h. The blood collected was centrifuged at 13,200 rpm for 5 min. 

### 2.4. Plasma Sample Analysis

Plasma samples were analyzed to determine ginsenosides Rb1, Rd, and Rg3 using liquid chromatography–tandem mass spectrometry (LC-MS/MS) in accordance with our previously validated method [25]. The plasma sample (20 μL) was deproteinized with 40 μL of acetonitrile containing an internal standard of 50 ng/mL. The sample was vortexed strongly and centrifuged at 15,000× *g* for 5 min. The obtained supernatant was transferred to LC vials, and a 5 μL aliquot was injected into the liquid chromatography–tandem mass spectrometry analysis system. 

The liquid chromatography–tandem mass spectrometry (LC-MS/MS) system consisted of an Agilent 1260 Infinity HPLC system with an Agilent 6460 triple-quadrupole mass spectrometer (Agilent Technologies, Palo Alto, CA, USA). The column utilized for separation was a Fortis C8 (2.1 × 100 mm, 5 μm, Fortis Technologies Ltd., Neston, UK) and was kept at a consistent temperature of 40 °C with a column oven under thermostatic control. Details of the analytical methods are described in our previous publication [26]. The following precursor/product ion pairs were monitored for electrospray ionization with a multiple-reaction monitoring analysis: ginsenoside Rb1 (1131.4–365.3), ginsenoside Rd (969.5–789.4), ginsenoside Rg3 (829.5–789.4), and digitoxin (969.5–789.4) in negative ion mode.

For calibration, stock solutions of ginsenosides Rb1, Rd, and Rg3 were dissolved in methanol to a concentration of 1 mg/mL. Working standard solutions were prepared by diluting the stock solution with methanol. The final concentrations ranged from 0.01 µg/mL to 10 µg/mL. Calibration standards were prepared by spiking 10 μL of working standard solution into 90 μL of blank mouse plasma at a final concentration range of 1–1000 ng/mL. Prior to analysis, the calibration standards were treated as described above. Calibration curves were constructed using a linear least squares regression of the analyte peak area ratio to the internal standard versus the analyte concentration. The regression coefficient (r^2^) was greater than 0.99. The accuracy and precision of the calibration standard curves were reliable for all tested concentration points. 

### 2.5. Metagenome Analysis

For metagenome analysis, fresh mouse fecal samples (0.2 g) were collected 24 h following the final delivery of amoxicillin via gavage. Genomic DNA was extracted from fecal samples using the method described by Kim et al. [19]. The V3–V4 region of the 16S rRNA was amplified and used for metagenomic analysis. Illumina Miseq was used for synthesis sequencing, and strains were determined via the EzTaxon-e database (http://eztaxon-e.ezbiocloud.net/ (accessed on 7 July 2021)). A genetic analysis of enzymes expressed by the microbiota was carried out using the method described by Langille et al. [27]. Sequencing reads were uploaded as fastq files, and this project was deposited in the NCBI under the accession number PRJNA851488. The fecal bacterial enzyme fraction was produced in accordance with the method of Kim et al. [28]. 

### 2.6. Microbial Metabolic Activity

The gut microbiota’s metabolic activity on ginsenoside Rb1 was assayed in a reaction solution (0.5 mL) containing 0.1 mL of the mouse fecal suspension and 0.4 mL ginsenoside Rb1 (4 mM). The reaction solution was incubated at 37 °C for 12 h and extracted twice with ethyl acetate (1 mL). The ethyl acetate layer was then concentrated and dissolved in methanol. The amount of residual ginsenoside Rb1 was determined. 

### 2.7. Data Analysis

All data are presented as means ± standard errors. Non-compartmental pharmacokinetic parameters, including the maximum plasma concentration (C_max_), time to reach C_max_ (T_max_), and the area under the plasma concentration–time curve from time 0–last (AUC_0-last_) for ginsenosides Rb1, Rd, and Rg3 were determined using the Phoenix WinNonlin Version 5.3 (Pharsight Corporation, Gary, NC, USA). All data were expressed as mean and standard deviation (SD) scores, and statistical significance was analyzed using a one-way ANOVA, followed by graphical output and Student’s t-test analysis using Graph-Pad Prism 9 (GraphPad Software, Inc., San Diego, CA, USA). The differences were deemed statistically significant at *p* < 0.05.

## 3. Results

### 3.1. Effect of Amoxicillin on the Gut Microbiota Composition

**Figure 1:**
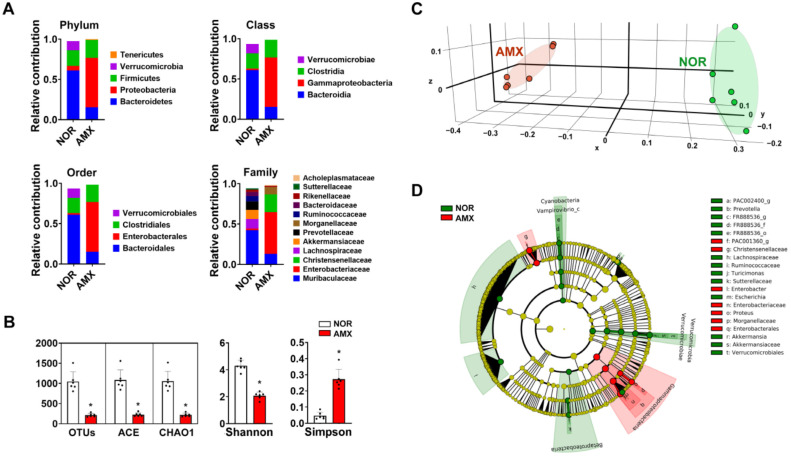
The effect of amoxicillin (AMX) on the microbiota of the gut. (**A**) The effect on gut microbiota composition. (**B**) The effect on alpha diversity. (**C**) The principal coordinate analysis (PCoA) plot using UniFrac. (**D**) The cladogram derived via a linear discriminant analysis effect size (LEfSE) analysis. Data expressed significant differences in gut microbiota composition up to the genus level between the normal control group (NOR, green) and the AMX-treated group (AMX, red). The threshold on the logarithmic LDA score for discriminative features was set at 4. Data are shown as means ± SDs (*n* = 6). * *p* < 0.05 vs. NOR.

The composition of the gut microbiota can be altered through exposure to antibiotics like amoxicillin [29]. To understand the impact of the gut microbiota on ginsenoside metabolism, we induced dysbiosis in the gut microbiota of mice using amoxicillin and investigated their gut microbiota composition. The oral gavage of amoxicillin led to a significant increase in gut microbiota composition at the phylum, class, order, family, and genus levels (Figure 1 and Table 1). In addition, amoxicillin treatment reduced the α-(OTU, ACE, CHAO1, and Shannon) and β-diversity (PCoA) of the gut microbiota (Figure 1B,C).

### 3.2. Effect of Amoxicillin on the Pharmacokinetics of Rg3

**Figure 2:**
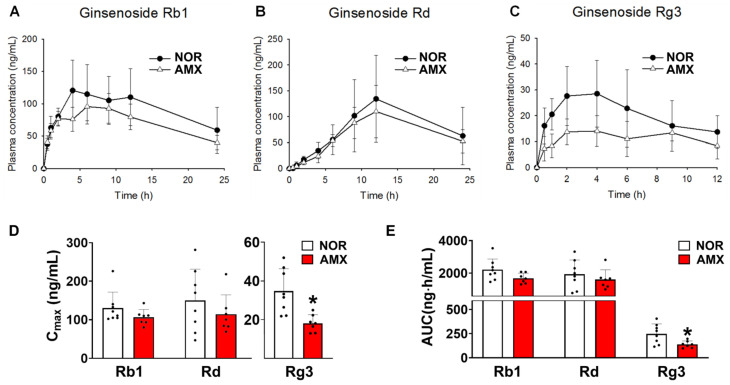
Effects of amoxicillin (AMX) on the pharmacokinetics of ginsenosides. (**A**–**C**) Plasma concentration profiles, (**D**) Cmax, and (**E**) AUC of protopanaxadiol ginsenosides Rb1, Rd, and Rg3 in mice treated with or without antibiotics. The red ginseng (10 g/kg) extract was orally administered to mice two days after the final amoxicillin dose. Amoxicillin was orally administered to mice once a day for five days before red ginseng treatment. Data represent means ± SDs (*n* = 8). AMX, amoxicillin-treated group. NOR, normal control group. * *p* < 0.05 vs. NOR.

The plasma concentration–time profiles of ginsenosides Rb1, Rd, and Rg3 are shown in Figure 2A–C in control and amoxicillin-treated groups, with associated pharmacokinetic parameters given in Table 2. The AUC values for ginsenoside Rb1 in the control and antibiotics-treated group were 2211.6 ± 614.5 ng·h/mL and 1684.9 ± 290.1 ng·h/mL, respectively. There was no statistical significance between the AUC values of the two groups. The C_max_ values of ginsenoside Rg3 in the control and amoxicillin-treated groups were 34.7 ± 10.8 ng/mL and 18.1 ± 4.1 ng/mL, respectively (Figure 2D). The AUC values for Rg3 in the control and amoxicillin-treated groups were 247.7 ± 96.6 ng·h/mL and 139.2 ± 32.9 ng·h/mL, respectively (Figure 2E). The C_max_ and AUC of Rg3 in the amoxicillin-treated group were considerably lower than those in the control group. The C_max_ and AUC of ginsenoside Rd in the amoxicillin-treated group showed a slight decrease compared to the control group; however, this difference did not reach statistical significance.

### 3.3. Effect of Amoxicillin on the Gut Microbial Metabolism of Rg3

**Figure 3:**
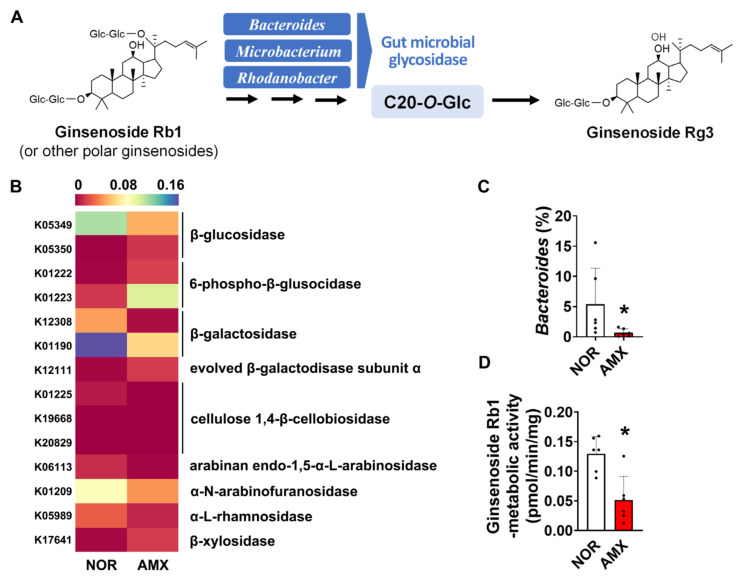
Effects of amoxicillin (AMX) on the microbial metabolism of ginsenosides. (**A**) Biotransformation of ginsenosides into ginsenoside Rg3 by gut microbiota. (**B**) The abundance of gut microbiome expressing the ginsenoside-metabolizing enzymes predicted using PICRUSt. Statistical significance was analyzed via the Kruskal–Wallis H test. (**C**) The abundance of Bacteroides based on synthesis sequencing analysis. (**D**) Effect of amoxicillin on ginsenoside-Rb1-metabolizing activity. Data are shown as means ± SDs (*n* = 6). AMX, amoxicillin-treated group. NOR, normal control group. * *p* < 0.05 vs. NOR.

Investigating the effect of amoxicillin on the ginsenoside biotransformation by gut microbiota, we analyzed the ginsenoside-metabolizing enzymes that the microbiota expressed by using a PICRUSt prediction of KEGG orthology pathways. The microbial enzymes investigated are including β-glucosidase, β-galactosidase, α-N-arabinofuranosidase, cellulose 1,4-β-cellobiosidase, arabinan endo-1,5-α-L-arabinosidase, and α-L-rhamnosidase, which may metabolize ginsenosides by catalyzing glycosidic bond cleavage. Amoxicillin treatment decreased the composition of microbiota expressing α-N-arabinofuranosidase, β-glucosidase, and β-galactosidase (Figure 3B). To evaluate the effect of amoxicillin on ginsenoside Rg3 formation, we also checked the abundance of *Bacteroides*, *Microbacterium*, and *Rhodanobacter*, which are known to be involved in the ginsenoside Rg3 formation (Figure 3A) [18] based on synthetic sequencing data. There was a significant decrease in the abundance of Bacteroides in the amoxicillin group (Figure 3C). However, *Microacterium* and *Rhodanobacter* were not detected in both groups. When the microbial ginsenoside-Rb1-metabolizing activity was measured, we observed that amoxicillin treatment notably decreased the ginsenoside-Rb1-metabolizing activity, reducing it from an average of around 0.13 pmol/min/mg to 0.05 pmol/min/mg. (Figure 3D). 

## 4. Discussion

In the present study, an amoxicillin-treated mouse model was used to examine how antibiotics impact the pharmacokinetics of ginsenosides following the oral administration of red ginseng extracts. Amoxicillin stands as one of the most frequently prescribed antibiotics and is widely utilized for addressing respiratory, skin, and urinary tract infections through treatment [30]. Considering its common usage and its known impact on altering the gut microbiome, we opted for amoxicillin as a typical representative of antibiotics in clinical practice and sought to explore the potential effects of combining amoxicillin with red ginseng. Thus, our research highlighted red ginseng as a feasible dietary supplement for patients managing various infectious diseases, thus prompting our interest in its interaction with amoxicillin.

We thought of amoxicillin as a commonly used antibiotic in clinical practice which causes an imbalance in the gut microbiome. On the other hand, red ginseng is essential for the metabolism of the gut microbiome, so we considered the combination of the two. Amoxicillin treatment induced a notable alteration in the composition of the gut microbiota (Figure 1); the result was a disruption of the microbial balance and a reduction in the richness and diversity of the microbial communities. A synthetic sequencing analysis showed that amoxicillin treatment led to an increase in the population of *Proteobacteria* and caused a decrease in the populations of *Bacteroidetes* and *Verrucomicrobia*.

The gut microbiota generates various enzymes possessing different metabolic functions. Specifically, enzymes that hydrolyze glycosidic bonds have been identified as having the ability to metabolize ginsenosides. Therefore, the changes observed in the makeup of the gut microbiota strongly indicate that the metabolic activities of gut microbial enzymes, including those involved in ginsenoside metabolism, are affected [18,23,25]. Metagenomic analysis data clearly demonstrate a significant suppression of microbial glycosidase enzymes, including β-glucosidase, and several enzyme gene expressions related to glucose-associated metabolism following amoxicillin treatment (Figure 3B). These findings provide support for the notion that the alteration of the gut microbiome profile caused by amoxicillin ultimately leads to changes in the biotransformation of ginsenosides, consequently affecting their pharmacokinetics.

As assumed, pharmacokinetic studies revealed a significant decrease in the systemic exposure of ginsenoside Rg3 following amoxicillin treatment subsequent to the oral administration of red ginseng extracts (Figure 2C). Although amoxicillin is known as a CYP2C8 inhibitor [31], our understanding suggests that CYP2C8 has a negligible role in ginsenoside metabolism. Furthermore, to eliminate any potential CYP-mediated interactions between amoxicillin and ginsenosides, red ginseng extracts were administered two days after the final amoxicillin treatment. Therefore, it is improbable that the changes in pharmacokinetics of ginsenoside Rg3 can be linked to the modulation of host metabolic enzyme activity by amoxicillin.

Our data indicate a significant reduction in microbial ginsenoside-Rb1-metabolizing activity in the amoxicillin-treated group (Figure 3D). This suggests that amoxicillin may diminish the gut microbial metabolism of ginsenoside Rb1, as well as other polar ginsenosides like ginsenosides Rb2 and Rc, and subsequently hinder the formation of ginsenoside Rg3 from these compounds. Consequently, this could explain the decrease in systemic exposure to Rg3 observed in the amoxicillin-treated mice. A study by Xu et al. [32] reported that ginsenoside Rb1 and its metabolites Rd and Rg3 were present in normal rat urine after Rb1 administration, whereas no Rb1 metabolites were detected in lincomycin-treated groups. This finding demonstrates that antibiotic treatment can influence the bacterial metabolic activities of ginsenoside Rb1 and cause alterations in the pharmacokinetics of its metabolites. These results align with our own findings.

Previous studies have reported the involvement of specific gut microbiota, including *Bacteroides*, *Bifidobacterium*, *Eubacterium*, *Flavobacterium*, *Fusobacterium*, *Lactobacillus*, *Microbacterium*, and *Rhodanobacter*, in the regulation of ginsenoside biotransformation [18]. These gut bacteria have a significant role in catalyzing the cleavage of glycosidic bonds at various positions in ginsenosides, leading to the removal of sugar groups. For instance, enzymes produced by gut bacteria such as *Bifidobacterium* and *Eubacterium* transform ginsenoside Rb1 into ginsenoside Rd. Furthermore, these bacteria are also responsible for converting ginsenoside Rd into ginsenoside F2 [18,23,25]. Similarly, the proportions of *Bacteroidetes*, *Eubacterium_g17*, and *Pseudoflavonifractor* were significantly reduced in the amoxicillin group, suggesting that they may be involved in the biotransformation of ginsenosides.

To generate ginsenoside Rg3, it is essential to cleave the glycosidic linkage at position C-20. This metabolic process is primarily attributed to *Bacteroides*, *Microbacterium*, and *Rhodanobacter* (Figure 3A) [18]. Our data demonstrated a significant suppression of *Bacteroides* following amoxicillin treatment (Figure 2C) [18,25]. This finding provides further support for the reduction in ginsenoside Rg3 generation through microbial biotransformation in amoxicillin-treated mice, in line with the observed changes in microbial β-glucosidase genes and ginsenoside-Rb1-metabolizing activity.

Significant interindividual variations in the pharmacokinetic profiles of red ginseng extracts have been documented. Choi et al. [33] reported a substantial standard deviation in the C_max_ and AUC values of ginsenoside Rd and compound K. Furthermore, research has shown that the gut microbiota may influence the pharmacokinetics of protopanaxadiol ginsenosides, including Rd, Rg3, F2, and compound K, in healthy individuals who received oral red ginseng treatment [25]. Therefore, our findings support the notion that the gut microbiome plays a part in observed individual variability in the pharmacokinetic profiles of ginsenosides when consuming red ginseng extracts.

## 5. Conclusions

In conclusion, amoxicillin treatment reduced both the α-diversity and β-diversity of gut microbiota and significantly altered the pharmacokinetic profile of ginsenoside Rg3 following the oral administration of red ginseng extract. The AUC of ginsenoside Rg3 decreased by approximately twice in the amoxicillin-treated group compared to the control. This reduction corresponded with the observed notable decrease in ginsenoside-Rb1-metabolizing activity due to amoxicillin treatment. Based on our findings, it appears that this change occurred due to modifications in the gut microbiota’s ability to metabolize ginsenosides, which happened as a result of alterations in the gut microbiota profile. Therefore, the difference in the gut microbiome profile could be the eventual cause of the individual variation in the therapeutic efficacy of red ginseng extracts. In this context, our results suggest that modulating the microbiome could enhance the efficacy of red ginseng. For example, some probiotics may be helpful for the gut microbial metabolism of ginsenosides and enhance the systematic exposure of bioactive non-polar ginsenosides. In addition, these findings offer valuable insights for advancing personalized therapeutics or personalized nutrition strategies. 

## Figures and Tables

**Table 1:** The ratio of the gut microbiota composition at the family, genus, and species levels in control and amoxicillin-treated mice.

Gut Microbiota		Composition (%) ^a^	
		Control	Amoxicillin
Phylum	*Bacteroidetes*	61.04 ± 13.05	15.26 ± 15.49 *
	*Cyanobacteria*	1.96 ± 2.07	NQ *
	*Firmicutes*	19.40 ± 8.50	22.18 ± 10.79
	*Proteobacteria*	5.78 ± 2.38	61.36 ± 5.73 *
	*Tenericutes*	0.19 ± 0.13	1.16 ± 2.12
	*Saccharibacteria_TM7*	0.08 ± 0.08	NQ *
	*Verrucomicrobia*	11.45 ± 9.46	0.01 ± 0.01 *
Class	*Bacteroidia*	61.02 ± 13.05	15.26 ± 15.50 *
	*Betaproteobacteria*	1.82 ± 1.76	NQ *
	*Clostridia*	18.93 ± 8.39	21.93 ± 10.95
	*Deltaproteobacteria*	0.93 ± 1.02	0.01 ± 0.01 *
	*Gammaproteobacteria*	2.09 ± 2.39	61.32 ± 5.73 *
	*Mollicutes*	0.19 ± 0.13	1.16 ± 2.12
	*Verrucomicrobiae*	11.45 ± 9.46	0.01 ± 0.01 *
Order	*Acholeplasmatales*	0.01 ± 0.01	1.15 ± 2.12 *
	*Bacteroidales*	61.00 ± 13.02	15.25 ± 15.51 *
	*Burkholderiales*	1.82 ± 1.75	NQ *
	*Clostridiales*	18.93 ± 8.39	21.93 ± 10.95
	*Desulfovibrionales*	0.92 ± 1.03	NQ *
	*Enterobacterales*	2.08 ± 2.39	61.31 ± 5.73 *
	*FR888536_o*	1.96 ± 2.07	NQ *
	*Lactobacillales*	0.26 ± 0.25	0.23 ± 0.26
	*Verrucomicrobiales*	11.45 ± 9.46	0.01 ± 0.01 *
Family	*Akkermansiaceae*	11.45 ± 9.46	0.01 ± 0.01 *
	*Bacteroidaceae*	5.48 ± 6.02	0.69 ± 0.64 *
	*Christensenellaceae*	0.38 ± 0.24	21.88 ± 10.94 *
	*Enterobacteriaceae*	2.05 ± 2.39	52.85 ± 5.86 *
	*Lachnospiraceae*	11.79 ± 5.69	0.03 ± 0.02 *
	*Muribaculaceae*	42.29 ± 8.52	12.98 ± 14.79 *
	*Prevotellaceae*	10.46 ± 3.41	0.01 ± 0.02 *
	*Rikenellaceae*	2.67 ± 0.59	1.34 ± 2.14
	*Ruminococcaceae*	6.38 ± 2.83	0.01 ± 0.01 *
	*Sutterellaceae*	1.81 ± 1.76	NQ
Genus	*Akkermansia*	11.44 ± 9.45	0.01 ± 0.01 *
	*Anaerotignum*	0.57 ± 0.42	NQ *
	*Bacteroides*	5.41 ± 5.92	0.69 ± 0.64 *
	*Clostridium_g6*	0.08 ± 0.06	0.00 ± 0.01 *
	*Enterobacter*	0.06 ± 0.08	49.87 ± 6.02 *
	*Enterobacteriaceae_g*	0.00 ± 0.00	1.63 ± 0.70 *
	*Escherichia*	1.99 ± 2.37	0.04 ± 0.04 *
	*Eubacterium_g17*	0.23 ± 0.19	NQ *
	*HM123997_g*	1.03 ± 0.85	NQ *
	*Muribaculaceae_uc*	0.96 ± 0.34	0.04 ± 0.06 *
	*Oscillibacter*	1.18 ± 0.98	NQ *
	*PAC000664_g*	2.05 ± 1.81	NQ *
	*PAC001360_g*	0.11 ± 0.05	19.90 ± 9.58 *
	*PAC002400_g*	3.74 ± 1.34	0.00 ± 0.01 *
	*Prevotella*	7.46 ± 2.85	0.01 ± 0.02 *
	*Pseudoflavonifractor*	1.66 ± 0.92	0.00 ± 0.01 *
	*Ruminococcus*	0.58 ± 0.50	NQ *
	*Turicimonas*	1.81 ± 1.76	NQ

Significant differences were assessed using the nonparametric two-tailed Mann–Whitney U test. ^a^ mean ± SD. * *p* < 0.05 vs. control group. NQ: less than 0.005.

**Table 2:** Pharmacokinetic parameters of ginsenosides Rb1, Rd, and Rg3 (*n* = 6).

Ginsenoside	Rb1		Rd		Rg3	
	Control	Amoxicillin	Control	Amoxicillin	Control	Amoxicillin
T_max_ (h)	8.8 ± 6.5	7.4 ± 2.5	13.5 ± 3.97	11.1 ± 1.4	2.8 ± 1.6	5.9 ± 2.8
C_max_ (ng/mL)	130.3 ± 38.5	106.3 ± 18.6	149.7 ± 76.2	114.1 ± 46.6	34.7 ± 10.8	18.1 ± 4.1 **
AUC (ng·h/mL)	2211.6 ± 614.5	1684.9 ± 290.1	1929.4 ± 823.2	1607.3 ± 551.7	247.7 ± 96.6	139.2 ± 32.9 **

** *p* < 0.01 vs. control group.

## Data Availability

Data are contained within the article and Supplementary Materials.

## Supplementary Materials

The following supporting information can be downloaded at https://www.mdpi.com/article/10.3390/foods12234342/s1, Figure S1: Representative HPLC chromatogram for the red ginseng extract used in this study.
