# Supplementary material for: Pharmacokinetic Profiling of Ginsenosides, Rb1, Rd, and Rg3, in Mice with Antibiotic-Induced Gut Microbiota Alterations: Implications for Variability in the Therapeutic Efficacy of Red Ginseng Extracts
Source: Foods. 2023 Dec 1;12(23):4342. doi: 10.3390/foods12234342 (PMC10706259; doi:10.3390/foods12234342)
Supplement: Supplementary file 1 [file foods-12-04342-s001.zip › foods-2709520-supplementary.pdf]

Pharmacokinetic Profiling of Ginsenosides, Rb1, Rd, and Rg3, in Mice with Antibiotic-Induced Gut Microbiota Alterations: Implications for Variability in the Therapeutic Efficacy of Red Ginseng Extracts

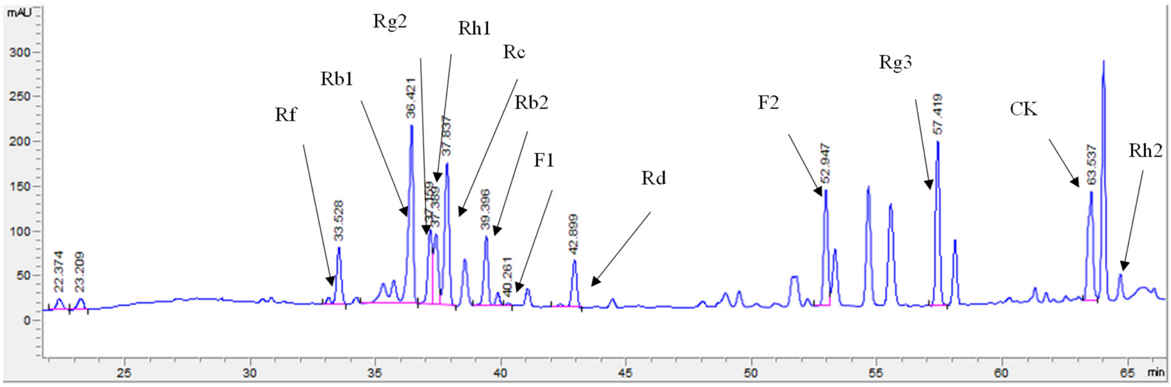

Figure S1. Representative HPLC chromatogram for the red ginseng extract used in this study.
